# Supplementary material for: Transcriptome profiling for precision cancer medicine using shallow nanopore cDNA sequencing
Source: Sci Rep. 2023 Feb 9;13:2378. doi: 10.1038/s41598-023-29550-8 (PMC9911782; doi:10.1038/s41598-023-29550-8)
Supplement: Supplementary file 1 — Supplementary Information 1. [file 41598_2023_29550_MOESM1_ESM.pdf]

SUPPLEMENTARY INFORMATION

Transcriptome profiling for precision cancer medicine using shallow nanopore cDNA sequencing

Andreas Mock[1,2,4], Melissa Braun[1], Claudia Scholl[3], Stefan Fröhling[1,2], Cihan Erkut[3,*]

[1] Division of Translational Medical Oncology, National Center for Tumor Diseases (NCT) Heidelberg, German Cancer Research Center (DKFZ), Heidelberg, Germany

[2] German Cancer Consortium (DKTK), Heidelberg, Germany

[3] Division of Applied Functional Genomics, DKFZ and NCT Heidelberg, Heidelberg, Germany

[4] Current address: Institute of Pathology, Ludwig Maximilians University Munich, Munich, Germany

* Corresponding author

Table S1. STAR alignment parameters

Parameter	Value
--alignIntronMax	1100000
--alignIntronMin	20
--alignMatesGapMax	1100000
--alignSJstitchMismatchNMax	5 -1 5 5
--alignSJDBoverhangMin	3
--chimJunctionOverhangMin	15
--chimScoreMin	1
--chimScoreJunctionNonGTAG	0
--chimSegmentMin	15
--chimSegmentReadGapMax	3
--clip3pAdapterSeq	AGATCGGAAGAGCACACGTCTGAACTCCAGTCA
--genomeLoad	NoSharedMemory
--limitBAMsortRAM	100000000000
--outBAMsortingThreadN	1
--outSAMstrandField	intronMotif
--outSAMtype	BAM Unsorted SortedByCoordinate
--outSAMunmapped	Within KeepPairs
--outFilterMismatchNmax	5
--outFilterMismatchNoverLmax	0.3
--outFilterMultimapNmax	1
--readFilesCommand	gunzip -c
--runThreadN	8
--sjdbOverhang	200
--twopass1readsN	-1
--twopassMode	Basic

Table S2. Minimap2 alignment parameters

Parameter	Value
-x	splice
-2	
-a	
-k	28
-I	100G
-w	30
-t	32
-K	1G
-L	
--cs	long
--sam-hit-only	
--MD	

Table S3. Run statistics for nanopore sequencing experiment. For each MinION (single or multiplexed) as well as Flongle runs, unique run ID, sample(s) included in the run, total number of reads, aligned reads, and bases sequenced, mean / median read length, N50, mean / median read quality and the corresponding basecall identity, as well as alignment accuracy (to the reference genome) are presented.

		MinION		MinION Multiplex		Flongle				
**Run ID**		*a94fd106*	*4591dc67*	*9f6b9859*	*06711035*	*59e4aae0*	*df143607*	*e2f013ab*	*2c45181e*	*c9dc0bab*
**Samples**		ACC1	ACC2	ACC3 ACC4 ACC5 ACC6	DDLS1 DDLS2 LCNC1 SS1	ACC1	ACC2	ACC3	ACC4	ACC5
**Number of reads**		3,110,122	2,252,388	1,246,685	2,131,066	30,544	53,185	8,552	9,617	14,891
**Number of aligned reads**		1,043,380	1,958,878	963,654	1,572,046	11,799	11,536	1,807	1,647	3,732
**Total bases sequenced**		4,283,949,966	3,390,114,748	1,911,633,148	2,773,898,959	35,747,708	75,397,147	9,920,502	7,500,025	15,997,046
**Read length**	*Mean*	1,377.4	1,505.1	1,533.4	1,301.6	1,170.4	1,417.6	1,160.0	779.0	1,074.3
	Median	998	1,139	1,190	973	895	1,076	805	635	788
	N50	2,030	2,115	1,961	1,717	1,577	1,994	1,672	952	1,448
**Read quality**	*Mean*	7.1	11.2	11.0	11.2	6.9	6.4	6.4	5.7	6.3
	Median	6.8	11.3	11.1	11.3	6.7	6.4	6.4	5.6	6.2
**Basecall identity**	*Mean*	80.5%	92.4%	92.1%	92.4%	79.6%	77.1%	77.1%	73.1%	76.6%
	Median	79.1%	92.6%	92.2%	92.6%	78.6%	77.1%	77.1%	72.5%	76.0%
**Alignment accuracy**		87.4%	87.6%	ACC3: 93.5%<br>ACC4: 93.6%<br>ACC5: 93.6%<br>ACC6: 93.5%	DDLS1: 93.4%<br>DDLS2: 93.4%<br>LCNC1: 93.8%<br>SS1: 93.8%	80.7%	87.4%	82.0%	83.8%	81.7%

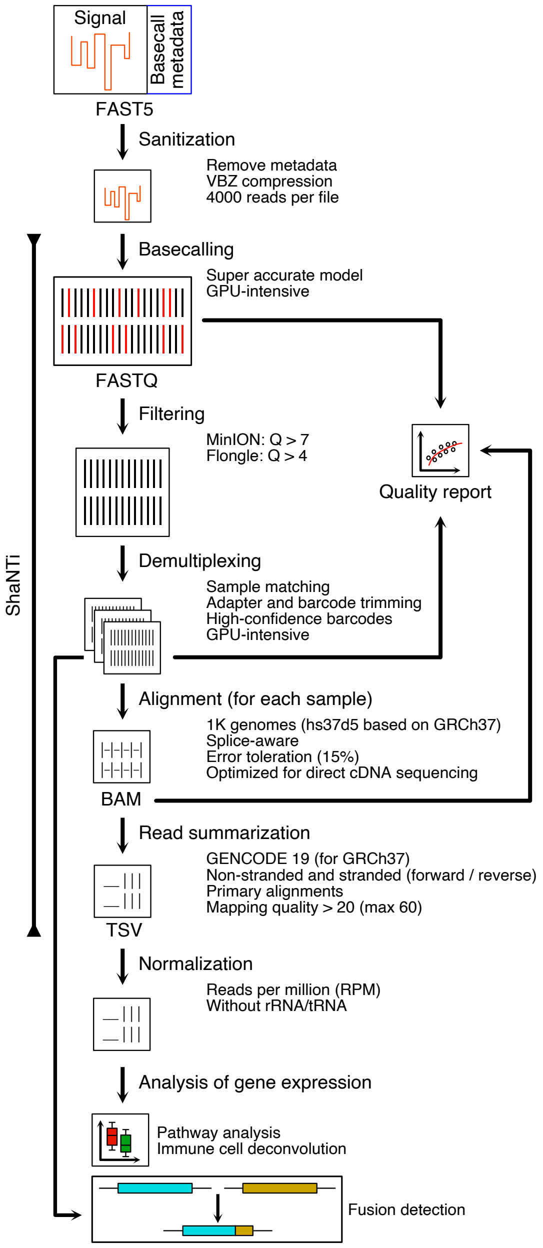

Figure S1. Data processing workflow for tumor transcriptome profiling by shallow nanopore RNA-seq. Current-level data are recorded in FAST5 files during sequencing. Metadata not needed for downstream analysis are removed, and signal data are compressed with the VBZ algorithm to generate standardized FAST5 files, which are used for basecalling with the “super accurate” model developed by Oxford Nanopore Technologies. The resulting sequence data are saved into FASTQ files. Low-quality reads based on the Phred score (indicated by red bars) are filtered out before further processing. For barcoded experiments, filtered FASTQ files are demultiplexed into individual samples and saved as separate FASTQ files. For each sample, reads are aligned to a reference genome, and the output is saved into sorted and indexed BAM files, which are used for read summarization, i.e., quantification of gene expression. The resulting counts are normalized to library size to yield calculated RPM values, which are used to analyze gene expression. In parallel, filtered and demultiplexed FASTQ files can be used to identify gene fusions employing a tool such as JAFFAL.

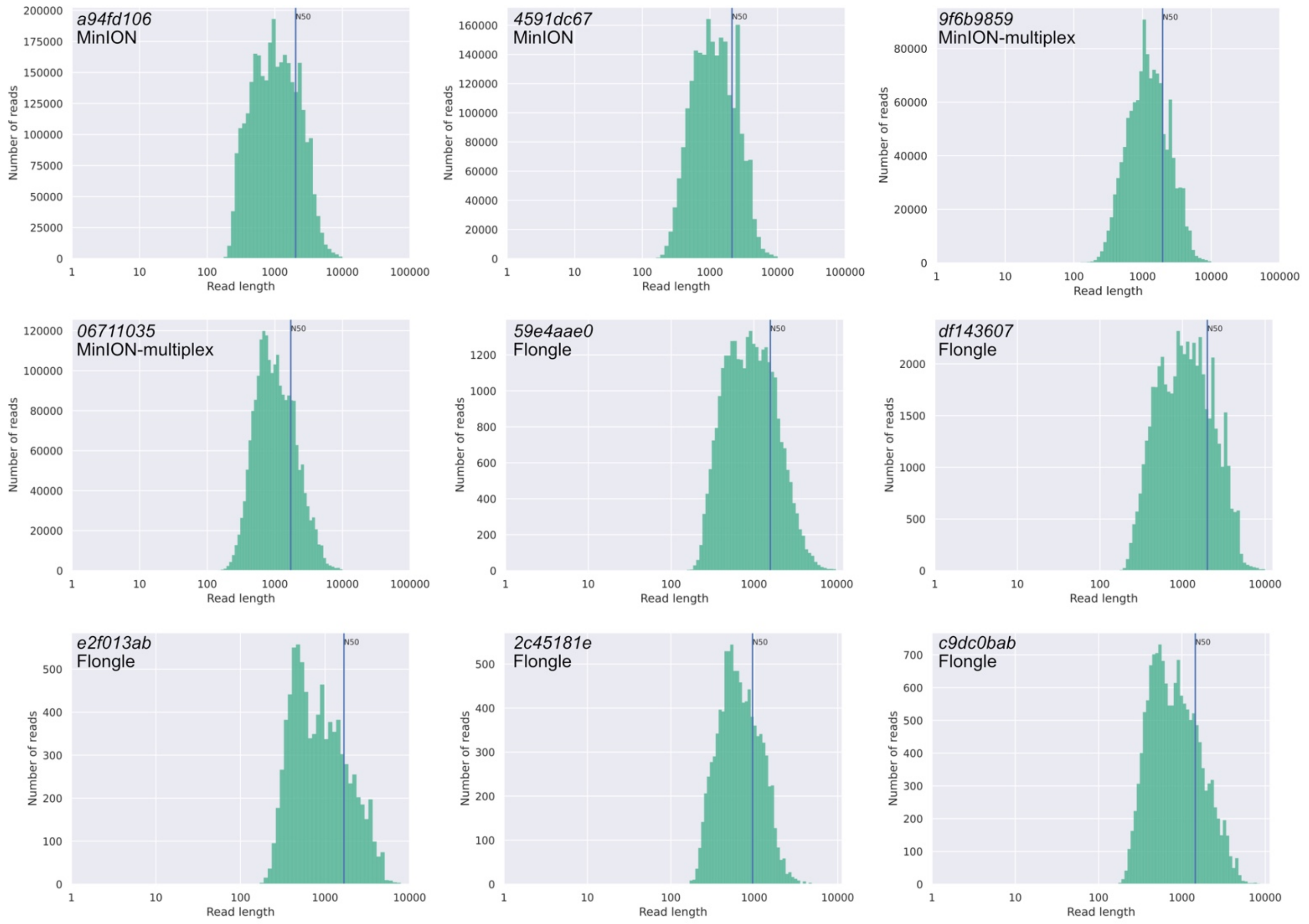

Figure S2. Read length distributions (log-transformed) for each MinION, MinION-multiplex and Flongle run. Unique Run IDs and the type of sequencing run are indicated on each plot and correspond to the run statistics on Table S3.

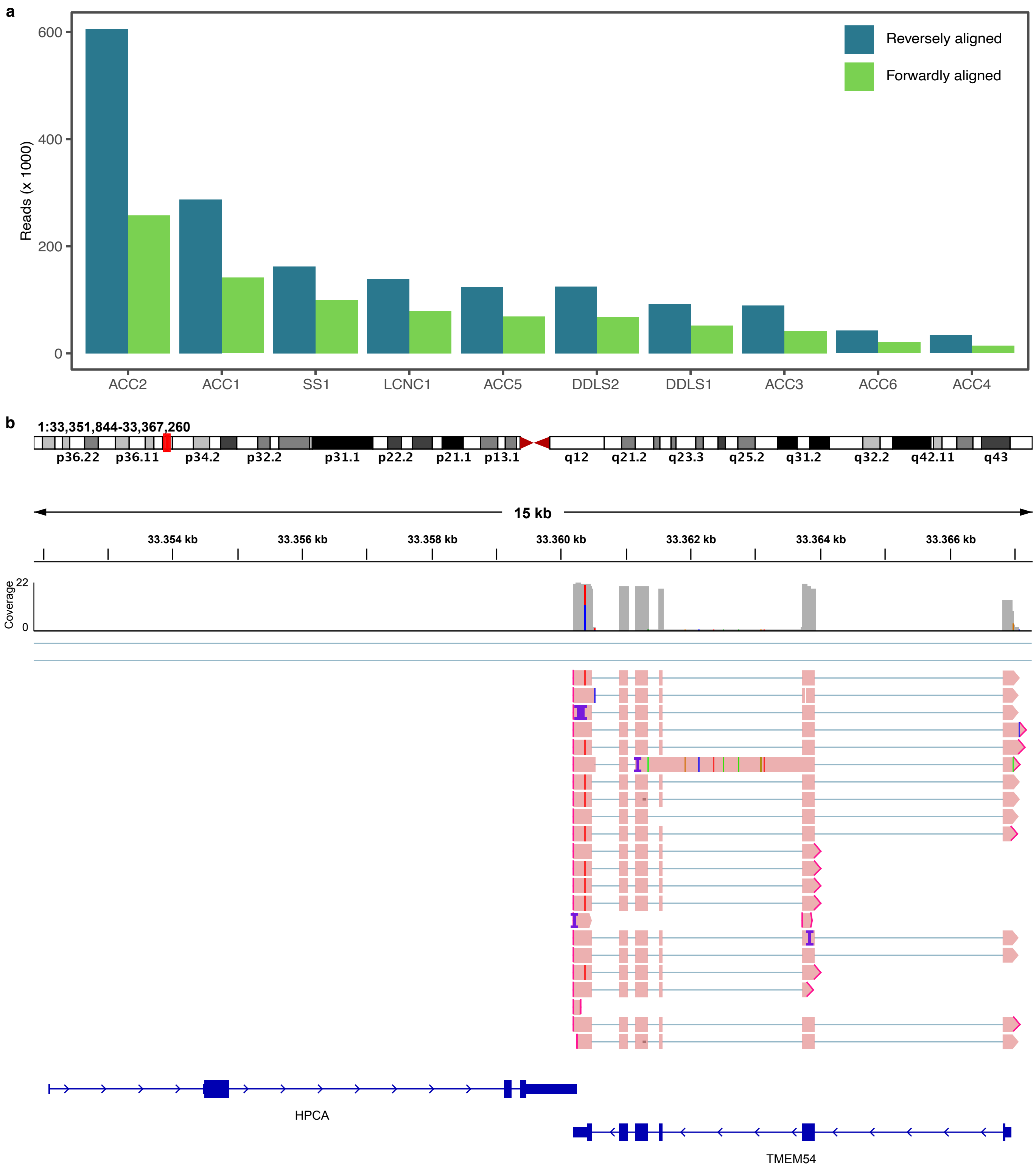

Figure S3. Effect of reverse transcription artefacts on direct cDNA sequencing data. a) Reverse transcription artefacts possibly cause an unbalanced distribution of first- and second-strand cDNAs. First- and second-strand cDNA reads align to the reference genome in reverse and forward orientation to gene models, respectively. We systematically observed around 2-fold more reversely-aligned reads (blue bars) than forwardly-aligned ones (green bars). **b)** For overlapping gene pairs, such as HPCA and TMEM54, the same reads are counted for both genes unless filtered for strand specificity, in which case 22 forwardly-and 22 reversely-aligned reads are reported for HPCA and TMEM54, respectively. A detailed look into the alignments, however, reveal that only TMEM54 transcripts are sequenced, which are all reversely-oriented.

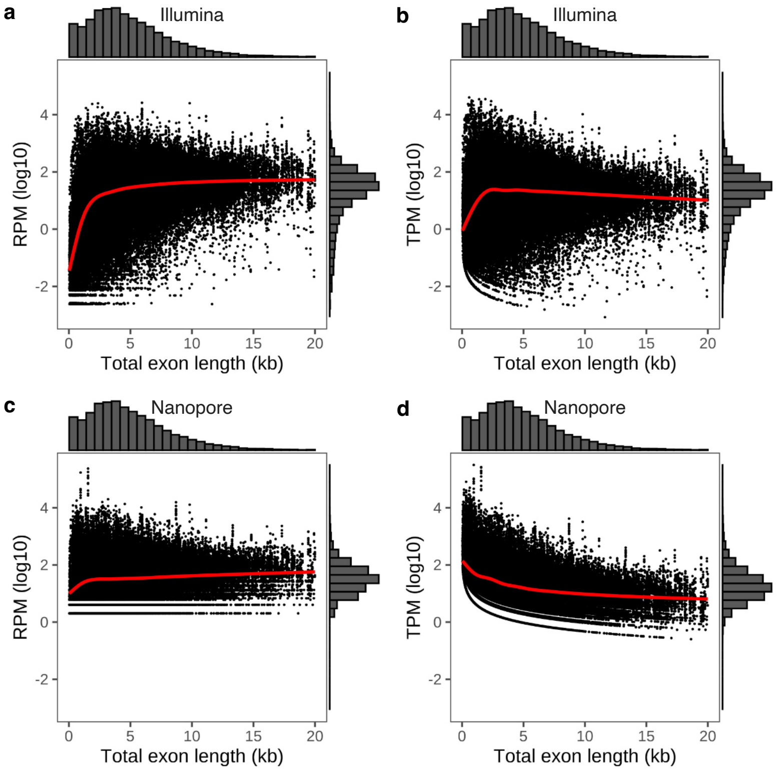

Figure S4. Visualization of the dependence of RPM and TPM metrics on total exon length. a) Reads per million (RPM) normalization, which is simply the ratio of reads counted for each gene divided by the total number of reads in the same library, multiplied by 1 million, shows a strong dependence on total exon length of the gene, especially for short transcripts. **b)** Transcripts per million (TPM) normalization, which normalizes reads to total exon length before to library size, alleviates this dependence. **c)** In contrast to Illumina, nanopore RPM metric is fairly independent of total exon length. **d)** Calculating TPM out of long read counts, however, introduces a (possibly non-linear) monotonic inverse relationship between TPM and total exon length. As a result of that, TPMs of longer transcripts are proportionally underestimated as compared to TPMs of shorter transcripts. In all panels, distributions of total exon lengths and log-transformed RPM/TPM metrics are shown as marginal plots.

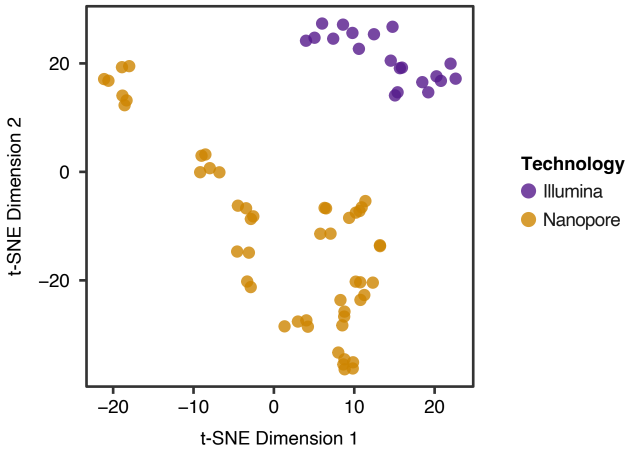

Figure S5. Visualization of TPM (Illumina RNA-seq) and RPM (shallow nanopore RNA-seq) values of all protein-coding genes using t-SNE.

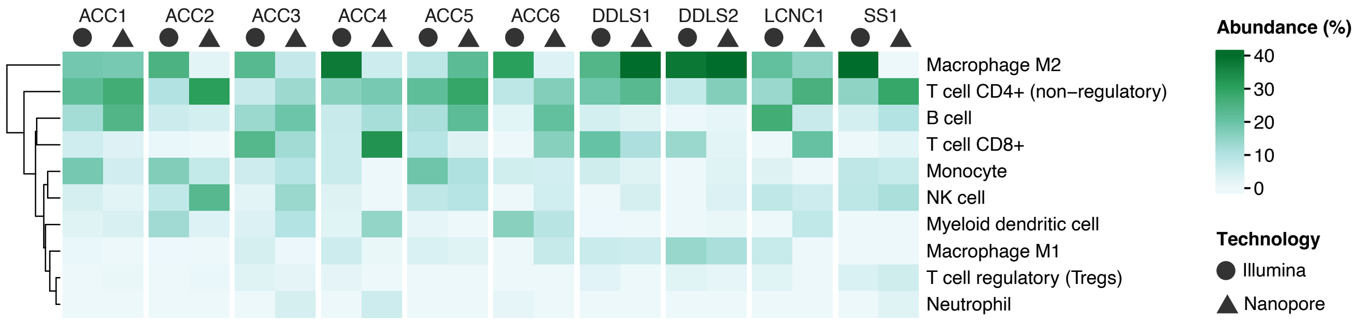

Figure S6. Immune cell abundance of ten tumor samples calculated with CIBERSORT based on Illumina and nanopore RNA-seq data.

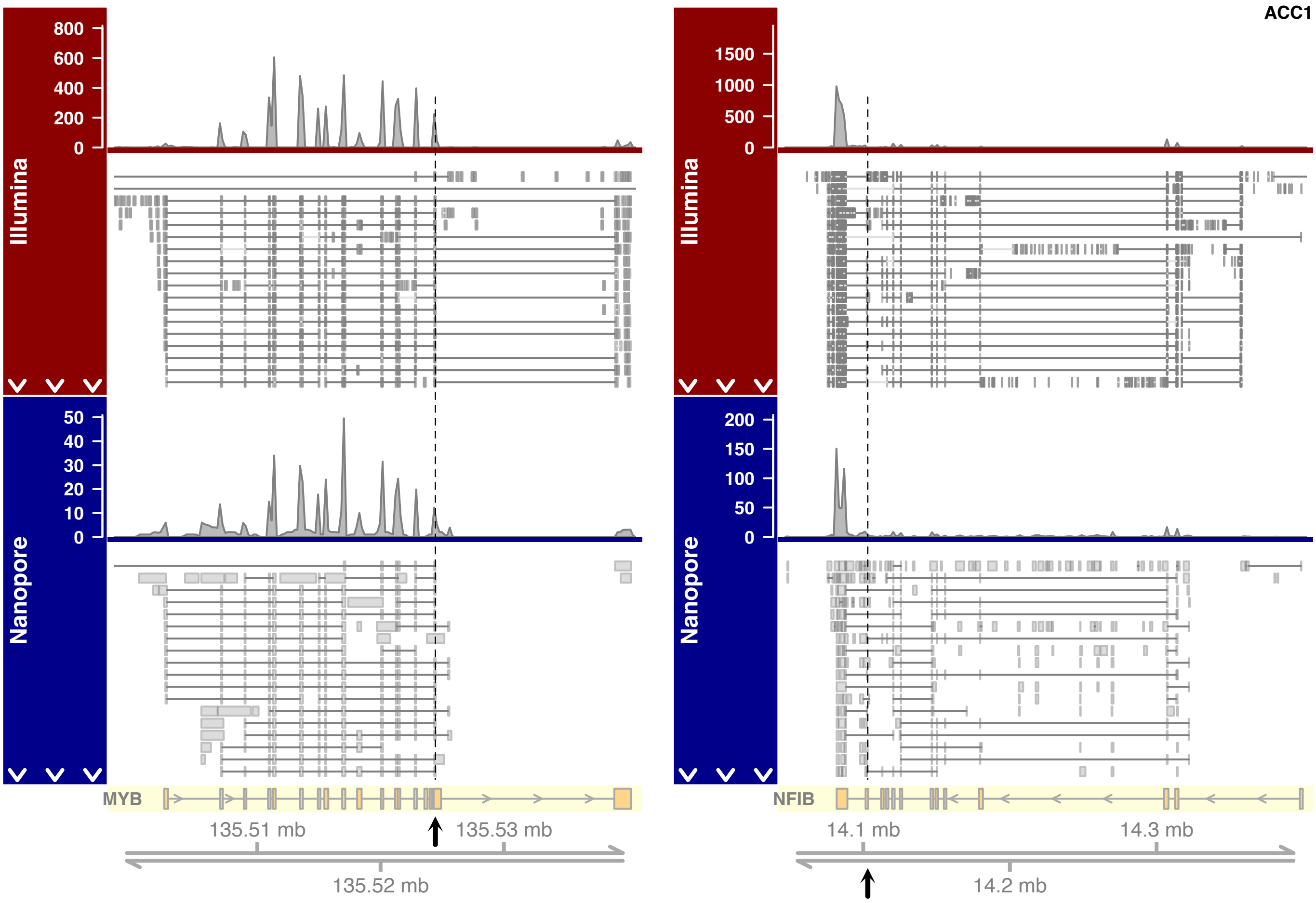

Figure S7. Visualization of short- and long-read alignments to *MYB* and *NFIB* in patient sample ACC1. Short (top) and long (middle) reads obtained from Illumina and shallow nanopore RNA-seq, respectively, of sample ACC1, plotted as alignments to the *MYB* (left) and *NFIB* (right) loci (bottom). For each read, aligned regions are represented by filled boxes, whereas spliced introns are shown as thin lines. The corresponding quantitative pileup results representing read coverage are plotted as bar graphs above the alignments. Black arrows indicate the position of the genomic breakpoint detected by whole-genome sequencing. In the representation of the gene model (bottom), arrowheads, boxes, and lines indicate the direction of transcription, exons, and introns, respectively.

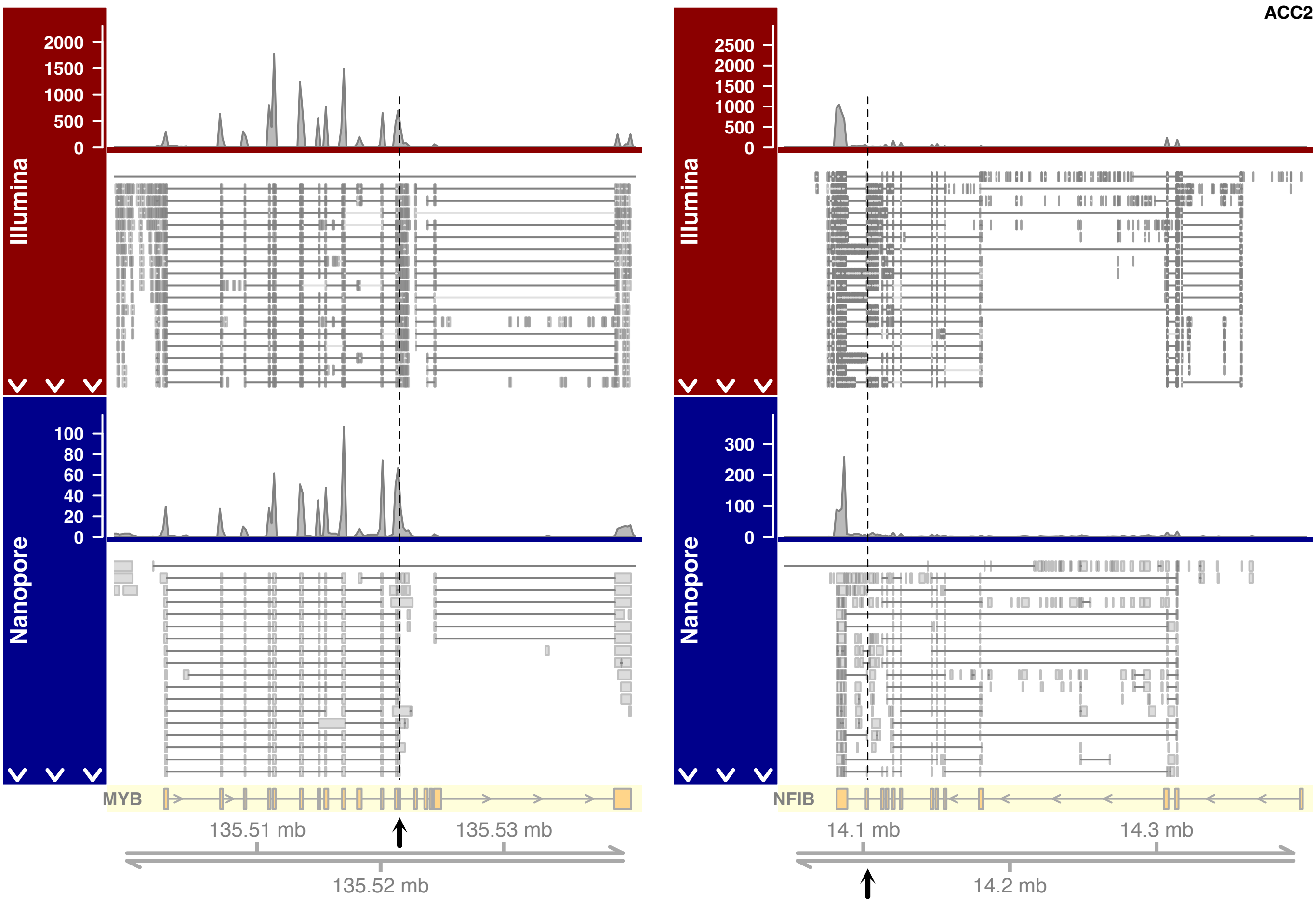

Figure S8. Visualization of short- and long-read alignments to *MYB* and *NFIB* in patient sample ACC2. See Figure S7 for description.

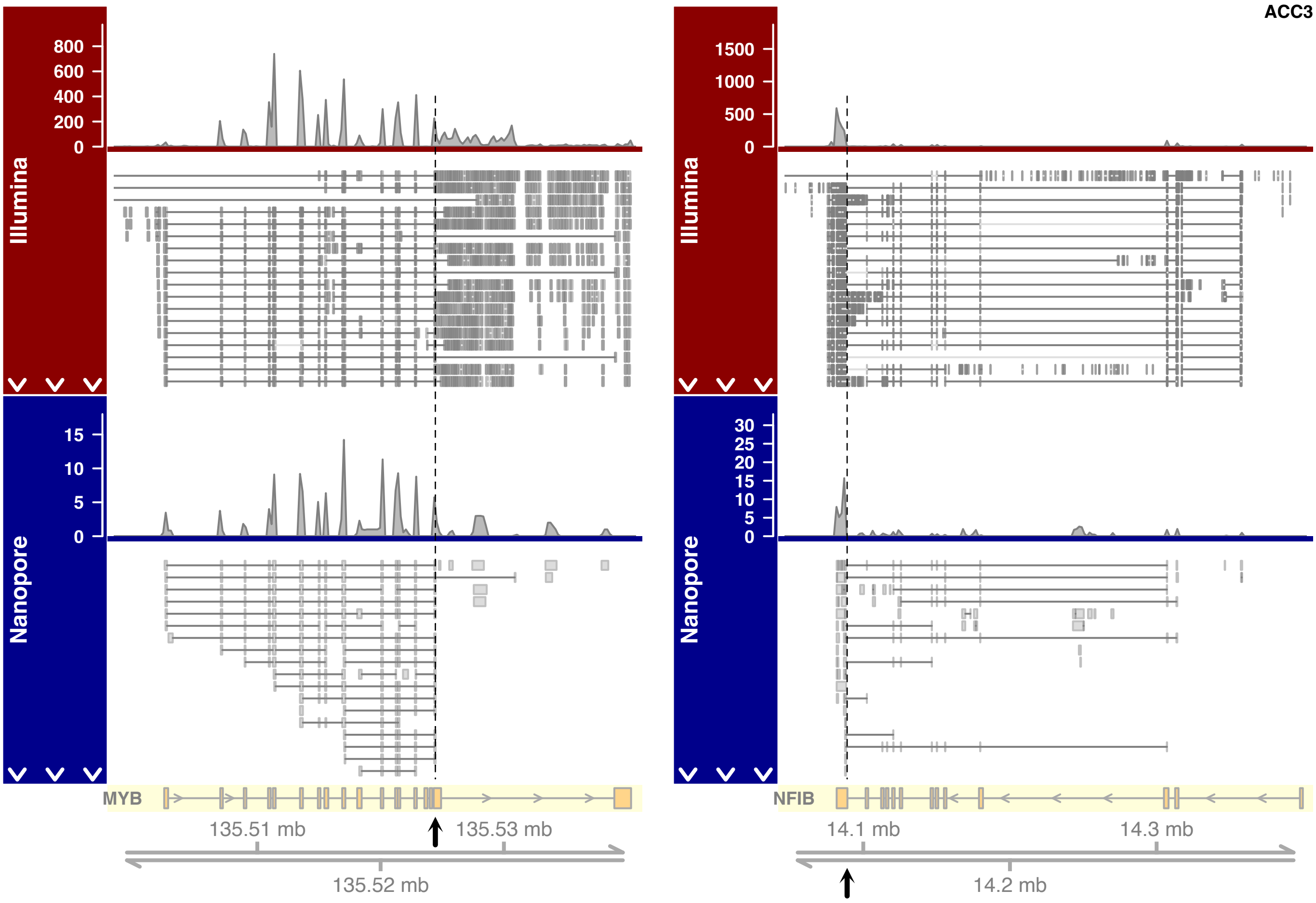

Figure S9 Visualization of short- and long-read alignments to *MYB* and *NFIB* in patient sample ACC3. See Figure S7 for description.

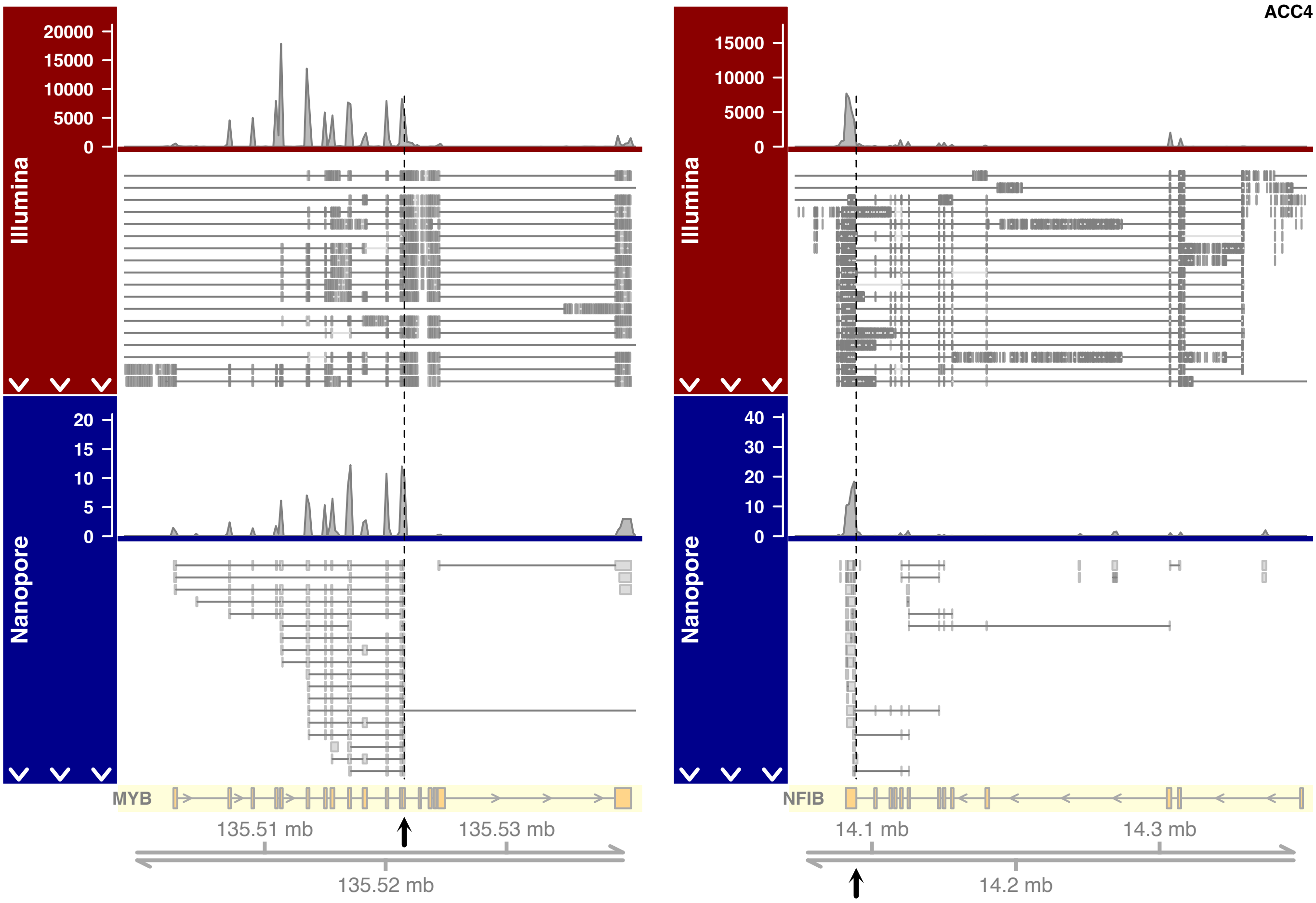

Figure S10. Visualization of short- and long-read alignments to *MYB* and *NFIB* in patient sample ACC4. See Figure S7 for description.

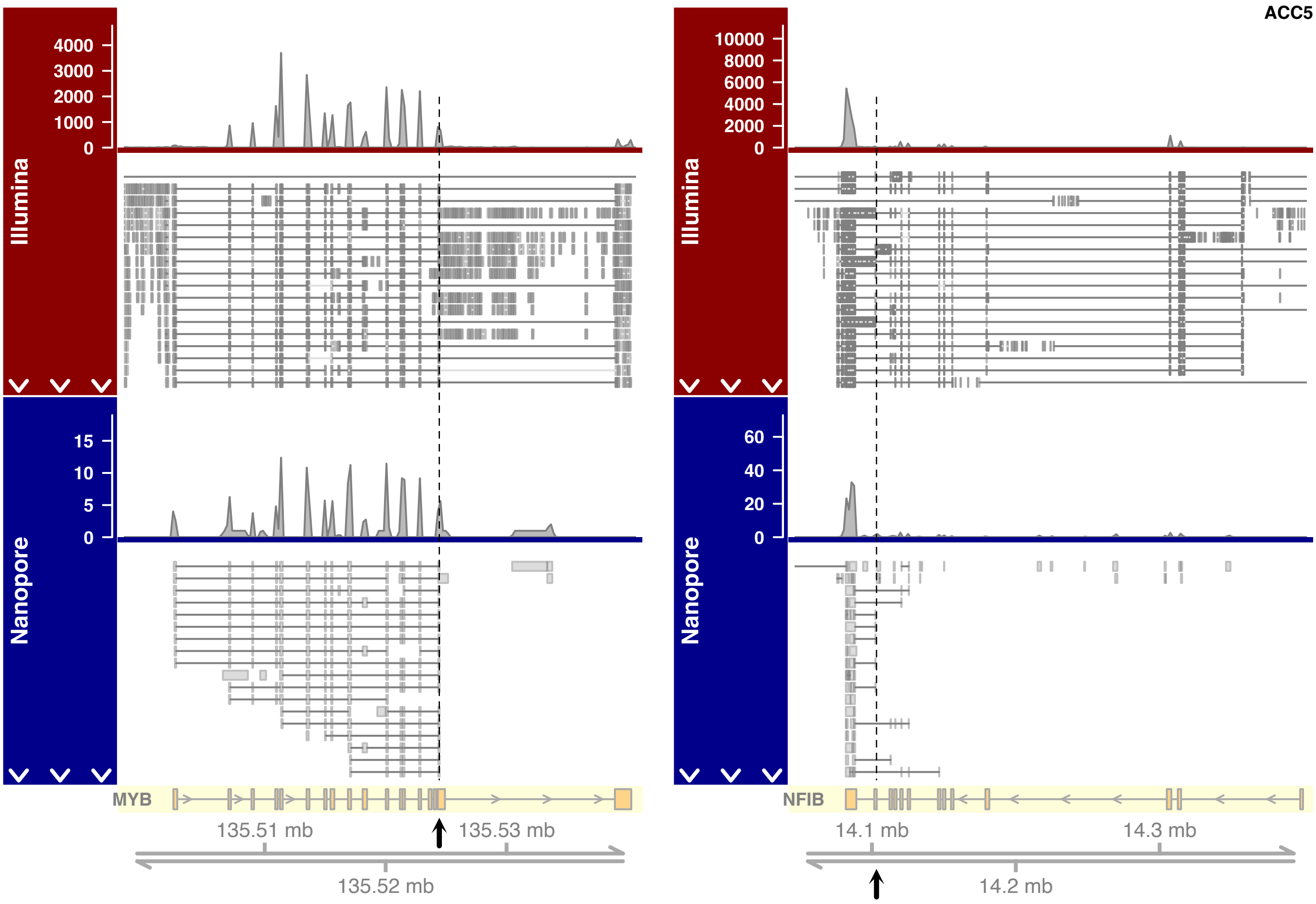

Figure S11. Visualization of short- and long-read alignments to *MYB* and *NFIB* in patient sample ACC5. See Figure S7 for description.

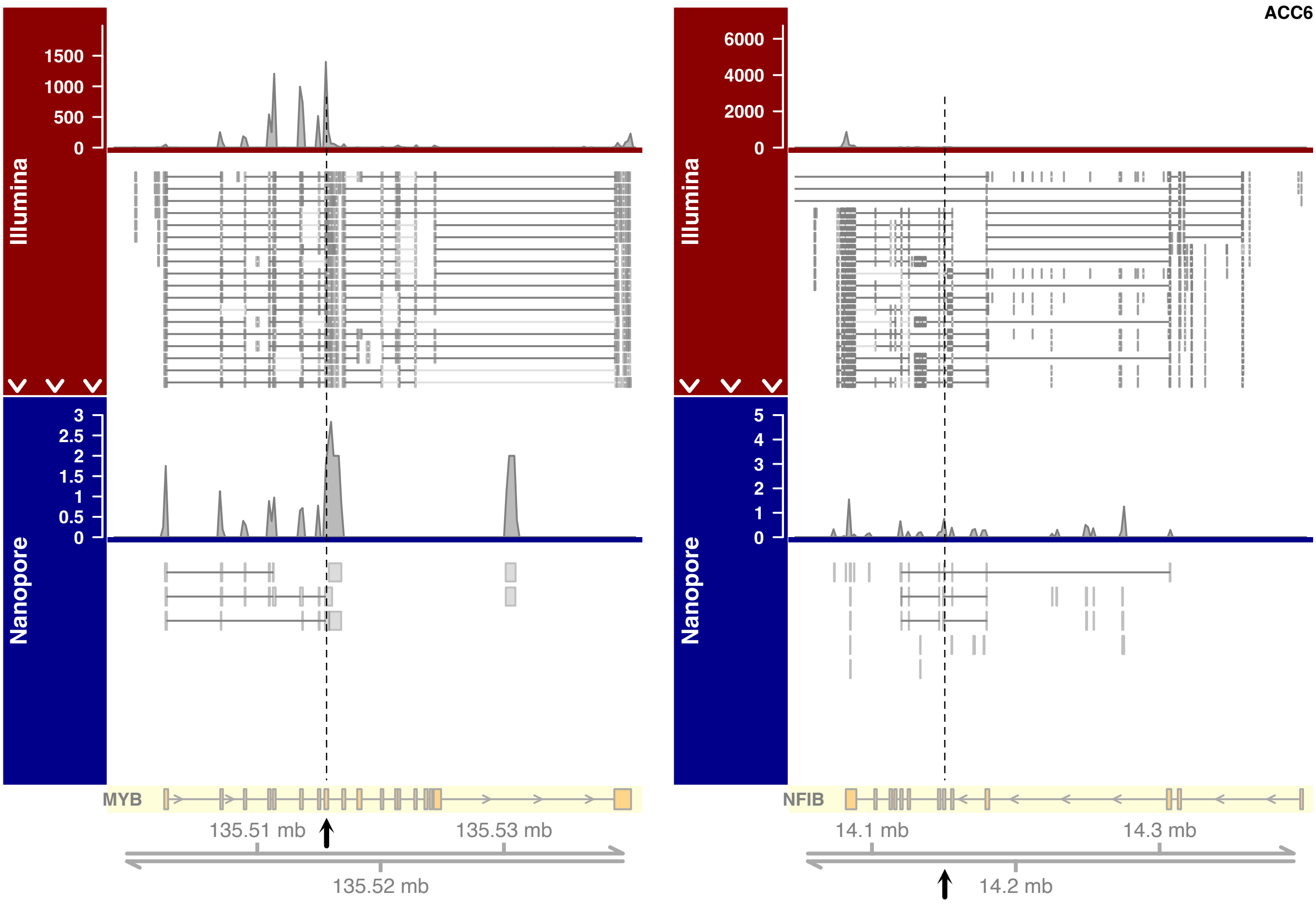

Figure S12. Visualization of short- and long-read alignments to *MYB* and *NFIB* in patient sample ACC6. See Figure S7 for description.

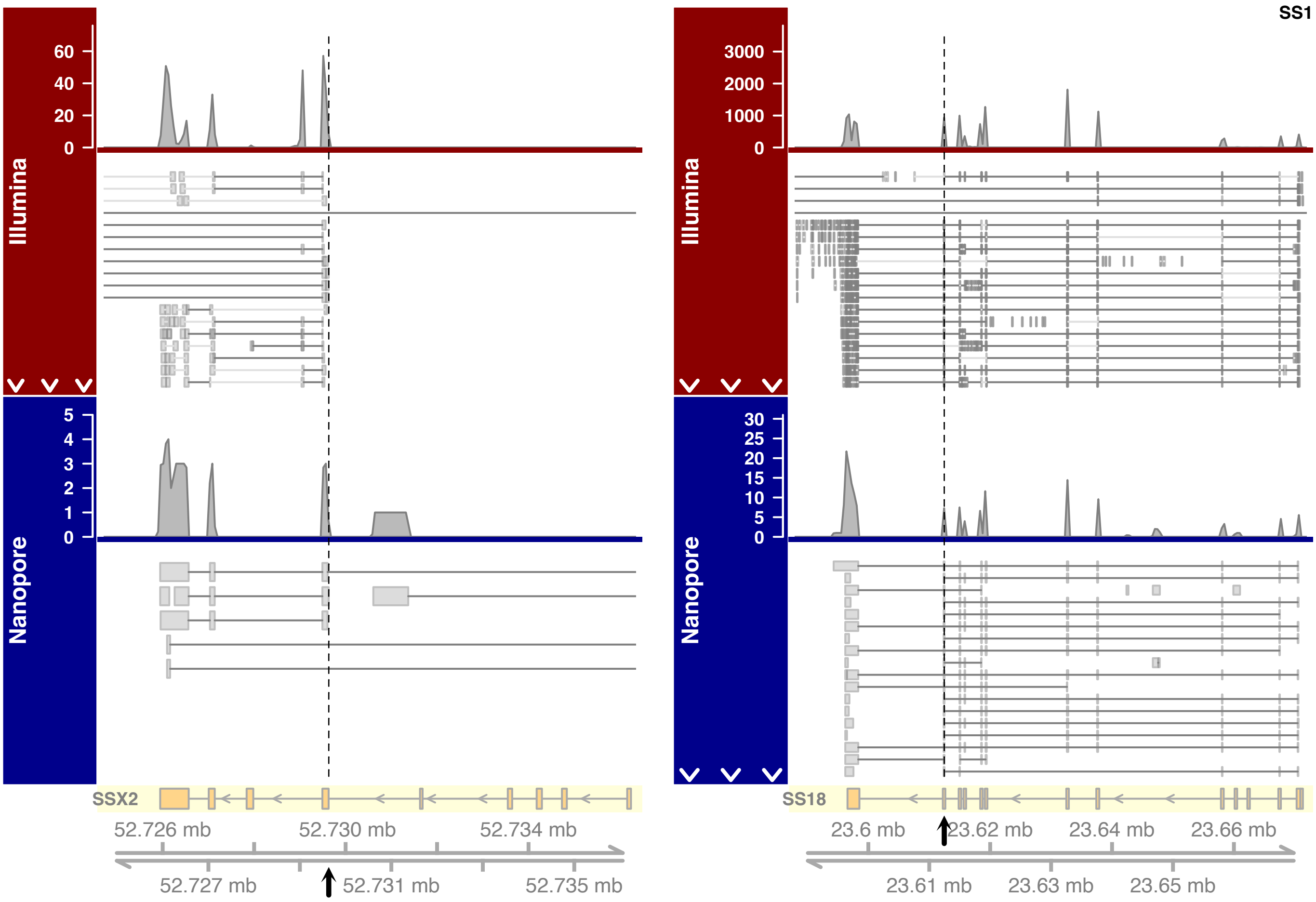

Figure S13. Visualization of short- and long-read alignments to *SSX2* and *SS18* in patient sample SS1. See Figure S7 for description.
